# Presumed Dissent? Opt-out Organ Donation and the Exclusion of Organs and Tissues

**DOI:** 10.1093/medlaw/fwac001

**Published:** 2022-02-16

**Authors:** Nicola J Williams, Laura O’Donovan, Stephen Wilkinson

**Affiliations:** Department of Politics, Philosophy and Religion, Lancaster University, Lancaster, LA1 4YW, UK

**Keywords:** Deemed consent, Opt-out organ donation, Organ donation policy, Policy exclusions and exemptions, Presumed consent, Transplantation ethics

## Abstract

It is often claimed that a legitimate approach to organ donation is an opt-out system, also known as ‘presumed consent’, ‘deemed consent’, or ‘deemed authorisation’, whereby individuals are presumed or deemed willing to donate at least some of their organs and tissues after death unless they have explicitly refused permission. While sharing a default in favour of donation, such systems differ in several key respects, such as the role and importance assigned to the family members of prospective donors and their preferences, and exclusions and safeguards which often specify the demographic groups, purposes, or organs and tissues that will remain outside the scope of the opt-out system. Using the recent shift to opt-out in England, Scotland, and Northern Ireland as case studies, and by reference to the key goals motivating this shift across the UK, this article asks whether and, if so, why, and how, opt-out systems for post-mortem organ donation should restrict the types of organs and tissues for which consent is deemed. In other words, ought opt-out systems for PMOD presume dissent regarding the donation of certain organs and tissues?

## I. INTRODUCTION

Within the philosophical and policy literature on organ donation, it is commonly claimed that a legitimate alternative to requiring explicit consent for organ retrieval from the deceased is an opt-out system, also known as ‘presumed consent’, ‘deemed consent’, or ‘deemed authorisation’.^1^ In such systems, the state presumes (or deems)^2^ that in the absence of explicit refusal of permission, individuals are willing to ‘donate’^3^ their organs and tissues post-mortem. Using evidence from psychology and the social sciences regarding ‘default effects’^4^ and procrastination and inertia among willing but unregistered organ donors,^5^ opt-out’s proponents claim that shifting the default in this manner is liable (in many nations) to both (i) increase the number of organs and tissues available for transplant, moving some way towards closing the transplant gap, and (ii) result in organ retrieval practices that better reflect the organ donation preferences of the majority.^6^ As a result of such claims—and despite objections from those who question the legitimacy of opt-out policies and/or their ability to provide the benefits their proponents promise—many countries have implemented opt-out legislation over the last 30 years,^7^ with England,^8^ Scotland,^9^ and the Netherlands^10^ joining this list in the last few years and Northern Ireland likely to follow later in 2022.^11^

While opt-out systems for post-mortem organ donation (PMOD) share a default in favour of organ donation, they differ in several key respects. One key example is the role and weight assigned to the views of the families of the deceased. Approaches to this range from those that afford family members of registered donors absolutely no power to influence PMOD outcomes (so-called ‘hard’ opt-out policies),^12^ to ‘softer’ ones that provide family members or nominated representatives with significant (sometimes overriding) power to shape donation outcomes.^13^ While the role and weight assigned to family views are important issues,^14^ this article considers a different and underexplored matter—the various *exclusions* built into opt-out systems. These normally specify groups for whom explicit consent is still required, with many countries excluding populations such as children, incapacitated adults, foreign visitors, and new residents.^15^ Such exclusions may also, and most importantly for the purposes of this article, be applied to specific types of organs and tissues. In this type of system, the kinds of organs and tissues for which consent is presumed are restricted, so that explicit consent is required from donors prior to death or, more likely, their relatives or nominated representative/s, post-mortem. In other words, dissent to particular organs and tissues falling within the scope of opt-out schemes is presumed. Examples of this can be found in the jurisdictions within the UK where a significant number of organs and tissues are excluded from the new (in Northern Ireland, proposed) opt-out arrangements on grounds of their being used for ‘experimental’ or ‘novel’ transplants.^16^

While such exclusions are commonplace, there has to date been little discussion of how they might be justified in either the philosophical or policy literatures on organ donation—a gap that this article seeks to address. By analysing the policy goals underpinning shifts to opt-out systems for PMOD in England, Scotland, Wales, and Northern Ireland, we aim to ascertain *whether* and, if so, *when, why, and how* opt-out systems for PMOD should restrict (and in the future revise) the types of organs and tissues for which consent can be deemed. In Section II, we begin by articulating the policy goals and arguments that motivated recent shifts to opt-out systems in England, Scotland, and Wales and the proposed shift in Northern Ireland. Section III then considers the relevance of those goals to questions about the *scope* of opt-out systems for organ donation, with a focus on restrictions placed on the kinds of organs and tissues included within such policies. Sections IV and V then examine and critique the policy development processes in England, Scotland, Wales, and Northern Ireland and make recommendations for improvement.

## II. BACKGROUND: POLICY GOALS UNDERPINNING THE SHIFTS TO OPT-OUT SYSTEMS FOR ORGAN DONATION ACROSS THE UK

### A. Increasing the Supply of Organs and Tissues for Transplantation

In philosophical, behavioural economics, and social psychology literatures surrounding organ donation, opt-out defaults are often proposed as a means of increasing the supply of organs and tissues for transplant (thereby reducing the so-called ‘transplant gap’) in nations with opt-in policy defaults for organ and tissue donation. While ‘default effects’ are not fully understood, opt-out defaults in organ donation are thought to increase supply for a number of reasons. Foremost among these is that opt-out defaults are thought to reduce the effects of procrastination and inertia in willing organ donors who ‘fail to get around’ to registering a positive preference to donate on an organ donor register (ODR).^17^ They are also thought to increase levels of donation willingness in the population more generally, by harnessing the effects of cognitive biases such as status quo bias, loss aversion, and implicit endorsement. These push the choices of prospective donors in the direction of the policy default (donation in the case of opt-out policies).^18^

While it may be reasonable to infer from the scientific and economic literatures surrounding choice architecture and defaults that opt-out systems are liable to increase donation rates when compared to opt-in systems, empirical evidence proving their efficacy in this respect is difficult to assess. Comparative data exploring donation rates across opt-in and opt-out nations,^19^ and within opt-out nations pre- and post-transition,^20^ do generally support claims regarding increased donation rates under opt-out policies. However, the utility of these is limited due to a range of confounding factors. Across nations, for example, mortality rates from road traffic accidents, overall health expenditure, religion, education, and transplant infrastructure significantly influence organ donation rates.^21^ Within nations, where increases have been observed after shifts to opt-out policies, ‘many other changes were introduced … such as better infrastructure or increased funding for transplant programmes’.^22^

Despite uncertainty regarding the effectiveness of opt-out PMOD systems in this respect, a key stated goal of shifts to such systems, has, in many nations, nevertheless been to increase the supply of organs and tissues available for transplantation.^23^ This has been the case in the UK, with policy and consultation documents in all four jurisdictions clearly articulating this goal. The Welsh 2011 Consultation and 2017 Impact Evaluation Report, for example, state that the aim of the shift to an opt-out system was to ‘increase the number of organ and tissue donors in Wales, allowing more lives to be saved’^24^ and to ‘increase the number of organs and tissues available for transplant’.^25^ English policy documents regarding the 2019 Organ Donation (Deemed Consent) Act explain that reforms were ‘intended to … increase the annual number and quality of organs transplanted so that everyone requiring a transplant stands the best chance of receiving one’,^26^ noting an ambition to see ‘700 extra transplants a year, transforming 700 lives’ as a result.^27^

Similarly, in Scotland, policy and consultation documents surrounding the Human Tissue (Authorisation) (Scotland) Act 2019 note both a core aim of ‘increasing the number of organ and tissue donors’^28^ and an ‘aspiration that transplantation waiting lists will decrease and the demand for transplants will be met’,^29^ to meet the government’s long-term goal to ‘reduce the numbers of people in Scotland waiting for transplants or dying waiting’.^30^ Further rationales underpinning goals of increasing the supply of organs and tissues for transplantation can be seen in the policy memorandum accompanying the 2019 Act. These include expressions of hope that the new system for organ/tissue donation will ‘support a Healthier Scotland’,^31^ by ‘increasing healthy life expectancy’,^32^ allow ‘individuals to return to work who are otherwise unable to do so because of serious illness’,^33^ and increase quality-of-life by ‘relieving the stress, anxiety, and the financial burden’^34^ experienced by those with long-term illnesses and their families. In Northern Ireland too, goals to increase organ and tissue supply are confirmed in a 2020 public consultation document surrounding the 2021 Organ and Tissue Donation (Deemed Consent) Bill.^35^ As in Scotland, where financial and social benefits are anticipated as a result of increased donation and transplantation rates, the Department of Health in Northern Ireland expresses similar hopes that the health service will benefit not only from ‘the reduced cost of treating patients whose health has been improved’,^36^ but also by ‘releasing resources to provide treatments for patients suffering from other ill-health conditions’.^37^

### B. Reflecting Individual Preferences Regarding PMOD

Another key benefit claimed of opt-out systems is their potential to generate organ retrieval practices that better reflect individual preferences surrounding PMOD in countries where donor registration rates remain low despite high levels of public support for organ donation. Notable examples of such countries include the USA, where 90% of the population support donation but only 60% are registered donors;^38^ Australia, where despite majority support (circa 76%)^39^ for donation, only 34%^40^ are registered donors; and England, where (prior to recent legislative changes) despite high levels of public support for PMOD (around 80%),^41^ and 65% of citizens expressing a willingness to donate post-mortem, only 39% of citizens had signed the ODR.^42^ This discrepancy between reported PMOD willingness and donor registration is generally attributed to procrastination and inertia in prospective organ donors who fail to register their (positive) donation preferences.^43^ Thus, in countries where there is a mismatch between public sentiment and donor registration rates, it is often claimed that shifting the default position from 'no' to 'yes' will result in an organ donation system that better reflects individual preferences.^44^

Appeals to positive preferences regarding organ donation have been used to both motivate *and* justify shifts to opt-out legislation across the UK. All four jurisdictions refer to opt-out’s potential to result in retrieval practices that better reflect the organ donation preferences of the majority. In Wales, for example, a 2011 consultation paper followed a statement of fact regarding registrations to the ODR (31%), with an appeal to research suggesting that ‘many more people would like to join the register but have not yet done so’.^45^ It was thus implied, but not explicitly stated, that an opt-out system could remedy this. In England, more explicit claims regarding the potential of opt-out in this respect have been made. These have included claims that opt-out ‘will better reflect what we know already, that the vast majority of the public in England support making their organs available to help others in need’,^46^ and that because ‘the vast majority of people support organ donation … it is right that we change the law to better reflect this’.^47^

In Scotland too, opt-out has been positioned as a means to ‘ensure individuals who would want to donate are able to do so’,^48^ and to solve problems ‘of people not getting round to making their [donation] wishes known’.^49^ Finally, in Northern Ireland, in policy documents it was lamented that given that 80% of the Northern Irish population supports organ donation, but only 48% have recorded a decision to donate, ‘the ODR is not yet truly representative of the Northern Ireland population’s willingness to donate their organs and tissue after their death’.^50^ Opt-out has, therefore, been presented as a potential solution to this problem, ‘as a means of … better reflecting the levels of public support for organ donation’.^51^

## III. SETTING THE SCOPE OF OPT-OUT LEGISLATION EXCLUDED ORGANS AND TISSUES

Across the UK, opt-out systems for PMOD have been implemented and/or proposed in service of the two key goals outlined above: increasing the supply of organs and tissues available for transplantation, and better reflecting individual preferences surrounding PMOD. However, it is not enough simply to decide to implement *an* opt-out policy; it is also necessary to specify *the key characteristics of the system beyond the default choice it imposes*. Numerous and potentially difficult decisions must thus be made regarding the details of such systems, and any exemptions and safeguards. These include decisions regarding the role that should be afforded to the families of would-be organ donors when these conflict with the avowed or deemed preferences of the donor; whether certain demographic groups such as children, overseas visitors, new residents, and adults lacking capacity to make decisions about organ donation should be exempt from the scope of opt-out provisions such that explicit consent to donation from the donor or, more likely, a legally appropriate proxy is still required; and whether the new opt-out arrangements should apply straightforwardly to *all* potentially transplantable organs and tissues, or whether certain organs and tissues be excluded from its scope and so still require explicit consent.

How should such matters be decided? Absent compelling reasons to the contrary, such details should be driven by the rationale for the prior choice to implement an opt-out system with the aim of maximising the coherence and effectiveness of the overall policy framework within which it sits. Thus, policymakers designing the details of opt-out systems across the UK (and other nations with similar policy goals), should do so with two questions at the forefront of their minds. First, which version of opt-out is most likely to meaningfully increase the supply of organs and tissues for transplantation? Secondly, which version is most likely to align PMOD outcomes with individual preferences surrounding PMOD?

While the role and weight assigned to family views is an important issue,^52^ and so too are questions surrounding excluded demographic groups, the focus of this article is the under-explored matter of scope exclusions applying at the level of organs and tissues (e.g. organs and tissues for which dissent, rather than consent, is presumed or deemed). Thus, in this section, we focus on whether, why, and if so, how, the exclusion of specific organs and tissues from the scope of opt-out across the UK is likely to better align posthumous donation outcomes with individual preferences surrounding PMOD, than opt-out systems that are not restricted in this manner. We also consider how, through reflecting preferences, opt-out systems that exclude certain organs and tissues from the scope of opt-out may also better serve the goal of meaningfully increasing the supply of organs and tissues available for transplantation. This will then inform work undertaken in Sections IV and V, where the approach taken to determining organ and tissue exclusions across the UK are examined.

### A. Reflecting Donation Preferences

Across the UK, appeals to high levels of public support for organ donation have been used to motivate shifts to opt-out systems, with policymakers readily pairing claims regarding public sentiment (for example, ‘8 out of 10 people say they would want to donate their organs and tissue after their death’), with claims that a shift to opt-out will better reflect these sentiments (for example, opt-out ‘better reflects the position of the majority of people who would be happy to donate their organs and tissue when they die’).^53^ Despite this, evidence from national ODRs, national and regional surveys tracking PMOD willingness, and large and small scale studies in the humanities and social sciences, have demonstrated that individual preferences surrounding PMOD are more complicated than can be garnered by reference to general levels of donation willingness alone. Instead, just as general preferences regarding organ donation differ from person to person, individual donation preferences differ from tissue to tissue, with high levels of variation demonstrated dependent on the organs and tissues in question.

In the UK, for example, evidence from the NHS Blood and Transplant service (NHSBT) shows that although 85% of *registered donors* are willing to donate kidneys, pancreases, hearts, lungs, livers, and corneas post-mortem, 15% selectively refuse to donate at least one of these, with 10.1% refusing to donate their corneas.^54^ Evidence from a 2018 survey of knowledge, attitudes, and behaviour of 4,001 members of the German population regarding organ donation shows similar levels of variation, finding that 13% of willing donors would refuse to donate certain organs and tissues with corneas, hearts, and skin the most likely to be refused.^55^ Small and large scale studies of donation preferences in the social sciences also provide evidence of this variation, with a study of 445 US adolescents’ attitudes surrounding PMOD finding that among respondents who expressed willingness to donate their organs and tissues post-mortem (49.2% of total respondents), a significant proportion would selectively refuse to donate their eyes (32%), pancreas (13.8%), lungs (12.8%), or heart (9.9%).^56^

The above data explore variations in PMOD preferences surrounding commonly transplanted organs and tissues, such as hearts, lungs, and corneas, and show that only a minority (albeit sometimes a sizeable one) of donors are likely to refuse their donation. A number of studies also explore variation in preferences surrounding the donation of less commonly transplanted, novel, or experimental organs and tissues, such as hands, feet, skin, faces, and uteri. These data, while relatively sparse, suggest that significantly lower levels of donation willingness may be observed for such organs and tissues than for more commonly transplanted organs and tissues.^57^ For example, the results of a 2016 German survey of 755 medicine and economics students’ PMOD preferences showed that while more than 70% respondents were willing to donate their kidneys and livers, only around 30% were willing to donate a hand or a foot or a ‘large area of skin’.^58^ Similarly, a 2014 study looking to preferences and rationales for organ donation among 1,027 individuals in New Jersey showed that respondents were less willing to donate uteri, hands, and faces than hearts, lungs, kidneys, and corneas.^59^ A 2019 Gallup poll in the USA also points to variation in donation willingness regarding novel transplants. For, while 90.4% of respondents to the poll supported or strongly supported PMOD, only 64% and 46.9% of respondents were willing to donate their hands and faces for transplantation post-mortem.^60^

Furthermore, research exploring selective refusals from donor families (and other proxies) to donate specific organs and tissues also provides evidence of significant variation in PMOD willingness dependent on the organs and tissues in question. In the UK, for example, data collected from consent forms signed by the families or nominated representatives of the 1,580 deceased organ donors in 2019–20 show that consent is more often provided for ‘major transplantable organs’ than for tissues. Refusal rates per organ and tissue among ‘consented’ donors were as follows: kidneys (0.25%), liver (0.38%), pancreas (1.08%), lungs (2.6%), heart (3.38%), bowel (5.71%),^61^ blood vessels (5.96%), heart valves (14.66%), skin (40.34%), bone (42.99%), tendons (49.21%), corneas (51.09), and meniscus (56.8).^62^ Similar data are available from Australia and New Zealand,^63^ and Brazil.^64^ Studies or reports of familial preferences with smaller sample sizes and/or a focus on particular organs and tissues also provide evidence of this variation. A study of 10,681 patient charts over a four year period in the USA, for example, shows significant differences between familial consent to PMOD generally (46.5%), tissue donation (34.5%), and corneal donation (23.5%),^65^ and a 2016 report from the UK’s ocular advisory group showed eye donation rates of only 40% among organ donors with family and donor refusals providing the reason for non-donation in 61.3% of cases.^66^

The studies and reports described here provide mixed evidence regarding the prevalence and content of selective PMOD preferences, where individuals exhibit willingness to donate certain organs and tissues post-mortem but not others. Unfortunately, little to no comparative research bringing together, comparing and/or exploring general trends arising from the data is currently available. However, what is clear is that while a shift to opt-out systems is likely to reflect public sentiment regarding the PMOD of certain organs and tissues, policymakers should not assume that this will be the case for *all* transplantable organs and tissues. Individual donation preferences can differ significantly from tissue to tissue, and the available evidence suggests that prospective donors are likely to be more willing to donate what might be thought of as commonly transplanted or life-saving organs and tissues, than those which may be used in experimental, novel, visible, uncommon, or non-life saving transplants, like skin, limbs, faces, bones, uteri.

Given this, where opt-out PMOD systems truly seek to reflect the preferences of prospective donors, concerted effort should be directed to uncovering donation preferences at the level of specific organ and tissue types prior to the implementation of the system, and to excluding organs and tissues for which levels of donation willingness are low. One cannot, after-all, in good conscience, appeal to positive public sentiments surrounding PMOD generally as justifying a shift to opt-out with respect to uterus, face, skin, or limb donation, when such sentiments are unlikely to apply to those organs and tissues.

Furthermore, public awareness of the ability to transplant some organs and tissues (for example, uteri, faces, or limbs) may be lower than for others, such as hearts, lungs, corneas, or kidneys. Given this, policymakers should be aware that in addition to lower levels of donation willingness with respect to such organs and tissues, a significant number of prospective donors may never have considered the possibility of their donation at all. Consequently, many prospective donors may well lack donation preferences altogether with respect to the donation for ‘novel’ or ‘experimental’ purposes, even in cases where they have signed an ODR and expressed a willingness to donate ‘any part’ of their body after death.^67^ In such instances, questions about the appropriateness of ‘deeming’ or ‘presuming’ consent to donation arise. These mirror concerns which may be raised regarding the appropriateness of including certain demographic groups within the scope of opt-out systems (such as children and adults who lack capacity, overseas visitors, and new residents). It would, after-all, be inappropriate to ‘presume’ or ‘deem’ consent to organ donation in those who lack the capacity to consent for themselves, and based on the inaction of those whom we cannot reasonably expect to be familiar with the mechanisms by which consent or refusal to organ donation is provided. Given this, it may also be argued that it would be unreasonable to ‘presume’ or ‘deem’ consent to the donation of organs and tissues for transplantation purposes so novel that very few individuals can be expected to have considered (and thus formulated donation preferences regarding) them.^68^

### B. Increasing Supply

Given the evidence regarding selective organ donation preferences explored above, and the lack of public awareness of organ transplantation, opt-out systems for PMOD which exclude organs and tissues on this basis are liable to result in donation outcomes which better reflect the population’s preferences (a core goal underpinning shifts to opt-out systems). Reducing the scope of opt-out could also help achieve the second core goal underpinning shifts to opt-out systems: meaningfully increasing the supply of organs and tissues for transplant. For, as explored below, excluding organs and tissues from the scope of deemed consent (presuming dissent or unwillingness) based on evidence about levels of donation willingness may ameliorate concerns that could otherwise lead previously willing donors to opt-out altogether.

While one of the major claimed advantages of opt-out systems is their potential to increase donation rates, critics of such systems also note their potential (given strongly held objections to opt-out policies for organ donation) to negatively affect public sentiment and erode trust in organ donation. This could result in significant numbers of potential donors (including some who would have signed the ODR under an opt-in system) opting out from PMOD.^69^ Notable objections to opt-out systems include beliefs that such systems fail adequately to respect the concept of organ donation ‘as a gift’, and associated worries that they increase the likelihood that organs will be retrieved from unwilling ‘donors’.^70^ Worries have also been expressed that opt-out systems pay insufficient attention to the preferences of family members of prospective organ donors, push the limits of legitimate state interference, bordering on or constituting ‘organ conscription’,^71^ or even increase the likelihood of ‘organ donation murder’ or the sub-par medical care of potential donors.^72^ Finally, it may also be argued that opt-out policy defaults are unjustifiably manipulative given their reliance on the effects of unconscious cognitive processes to increase donation rates. As explained in Section II, a key mechanism by which policy defaults are considered to increase donation rates is through harnessing the effects of unconscious cognitive biases such as status quo bias, loss aversion, and implicit endorsement to ‘nudge’ choices in a particular direction (towards the default). MacKay and Robinson, however, argue that this is ‘disrespectful of people's autonomy’^73^ as it takes ‘deliberate advantage of their cognitive biases … bypassing, not engaging their rational capacities’.^74^

Such concerns may or may not be well founded, but they could nonetheless have negative effects on donation rates, in some jurisdictions at least. In England, 15% (2,509 of 16,730) of respondents to the Department of Health and Social Care (DHSC)’s 2017 consultation on ‘Introducing “opt-out” consent for organ and tissue donation in England’ answered the question: ‘If the law changes would this affect your decision about organ donation’ with ‘yes, I will opt-out’.^75^ Should 15% of previously willing organ donors register their refusal to donate under an opt-out policy, this would significantly limit any gains in organ donation rates in England associated with opt-out. Another pertinent example is Brazil, where opt-out PMOD legislation was repealed a year after its implementation in 1998. *The Lancet* reported that ‘popular imagination’ played a key part in this:

Part of the population feared that their organs would be removed even before they were clinically dead. Many rushed to public offices to register themselves as non-donors, to avoid such a risk.^76^

As well as some people’s general concerns, specific incidents attracting adverse publicity could also suppress donation rates. A key example of this is the so-called Amiens Affair which occurred in France in the early 1990s. In this case, a legal complaint was filed by the parents of Christophe Tesniere, after the legally permitted but not explicitly parentally sanctioned removal of their son’s eyes during a PMOD procedure.^77^ This captured the public imagination, leading to significant reductions in public trust surrounding organ donation in France,^78^ and a fall in corneal donation rates (by 38% from 3,774 in 1991 to 2,383 in 1993) after the incident.^79^ Donation rates then took over four years to return to levels observed in 1991.^80^

The purpose of raising these concerns here is not to advance an objection to opt-out *per se*. Rather, the point is that even if a move to opt-out would be generally positive in terms of donation rates, there are some disadvantages (notably those linked to public trust) that could reduce any potential gains. Thus, in order to make opt-out systems as effective as possible, assuaging people’s concerns and so reducing the extent of those disadvantages is desirable. There are several ways of doing that. Clear and consistent messaging about precisely how opt-out works is one. Another way, more relevant for our purposes, is limiting the scope of opt-out so that *types* of transplantation liable to arouse discomfort or mistrust are left outside the scope of the opt-out system.

One relatively straightforward example of how this approach might work in practice is the choice between ‘hard’ and ‘soft’ opt-out systems, the latter being ones in which the deceased person’s relatives or other living third parties have a formal role in posthumous donation decision-making. On the face of it, ‘hard’ opt-out systems would seem best in terms of organ supply as they do not allow relatives to stand in the way of donation. However, such policies may 'backfire' by encouraging more people to opt-out during their lifetimes, fearful of a scenario where organs are taken against the wishes of their loved ones. Thus, an argument could be made that ‘soft’ opt-out is better, not only because of the respect it affords to relatives but also because it is more likely to maximise supply in the long term. The same kind of argument can be made in relation to the scope of opt-out systems, with respect to either tissue types or purposes. If rare, novel or experimental transplants were within scope of the opt-out, people who were worried that their bodies may be used in ways that they either fear or do not understand may be inclined to opt out completely. However, if opt-out arrangements only applied to familiar lifesaving transplants (such as kidneys, hearts, or livers) and decisions regarding organ and tissues excluded from the scope of opt-out were based on evidence regarding public preferences regarding PMOD, this could provide comfort to those with such worries. Those individuals may then feel less motivated to completely exit the donation system, provided that the limited scope of the opt-out system was clearly communicated.

Limiting the scope of opt-out polices, then, may well have advantages for the overall organ supply. It could also go some way towards addressing concerns about legitimate state interference in organ donation from those who believe that pushing, or nudging, as Thaler and Sunstein would have us call it,^81^ at such limits can be justified in certain ‘high stakes’ cases and contexts. This would include saving lives or in cases of organ shortage, but not others situations, such as cosmetic, experimental, or reproductive purposes, or where there is no shortage.

## IV. ORGAN AND TISSUE EXCLUSIONS ACROSS THE UK

Previous sections have outlined the more general rationales underpinning shifts to opt-out systems across the UK, and explored the potential for policy exclusions applied at the level of organs and tissues to support the goals of meaningfully increasing the supply of organs and tissues available for transplantation *and* better reflecting the preferences of potential organ donors. In this section, after setting out some foundational information regarding how opt-out legislation operates across the UK and the policy timeline, we examine the rationales underpinning organ and tissue exclusion policies across the UK. This is done with the aim of providing necessary context for the critical work following in Section V.

The legal basis underpinning organ donation in the UK is ‘appropriate consent’ in England, Wales, and Northern Ireland,^82^ and ‘express authorisation’ in Scotland.^83^ These types of consent are considered in place where an individual provides written consent to donation prior to their death, they are registered as donors on the national ODR, and/or where their nearest family members (that is, those in a ‘qualifying relationship’ to them)^84^ or nominated representative,^85^ authorise PMOD. Given that appropriate consent/express authorisation forms the cornerstone of the regulatory apparatus governing organ donation, for the shift to an opt-out system to become operational in England, Scotland, and Wales, legislative amendment of donation and transplantation laws was required to accommodate ‘deemed consent’ in England and Wales^86^ and ‘deemed authorisation’ in Scotland,^87^ and to specify the groups of persons to whom these apply.^88^ Alongside this, secondary legislation was required to specify the organs and tissues excluded from the opt-out systems, meaning that explicit consent or express authorisation to their donation will continue to be required—from the deceased, their nearest relative(s), or nominated representative (in England and Wales).^89^

### A. An Overview of the Legislative Timeline

The chronology of the UK’s legislative shift to opt-out began in Wales with the passing of the Human Transplantation (Wales) Act 2013.^90^ The new ‘deemed consent’ system did not become operational until December 2015 following the enactment of the Human Transplantation (Excluded Relevant Material) (Wales) Regulations 2015,^91^ which specified the meaning of ‘excluded relevant material’—material to which the 2013 Act does not apply.^92^ Almost four years later, in 2019, legislation setting out a statutory framework for opt-out was passed in England and Scotland. By virtue of section 1(4) of the Organ Donation (Deemed Consent) Act 2019, ‘deemed consent’ in England applies only to ‘permitted material’—material other than a type specified in regulations.^93^ In May 2020, the English opt-out system went live with the coming into force of the Human Tissue (Permitted Material: Exceptions) (England) Regulations 2020,^94^ which specify types of ‘relevant material that is not permitted material’.^95^ In Scotland, the Human Tissue (Authorisation) Scotland Act 2019 provides that ‘deemed authorisation’ does not apply in relation to an excepted body part,^96^ and following the passing of the Human Tissue (Excepted Body Parts) (Scotland) Regulations 2020,^97^ which set out relevant groups of excepted organs and tissues, the new system became operational in March 2021.

The role of the deceased’s family in opt-out systems should be noted. To determine whether consent to donation can be deemed, qualifying relatives of the deceased will be asked whether they have information that would lead a reasonable person to conclude that the potential donor would not have consented.^98^ If information to this effect is provided, then donation will not proceed. Nor will it proceed if no family is available to provide information about the deceased’s last known wishes. This is because the Human Tissue Authority considers that ‘the risks to public confidence of [donation] proceeding in these circumstances would outweigh the benefits’.^99^

**Figure 1:**
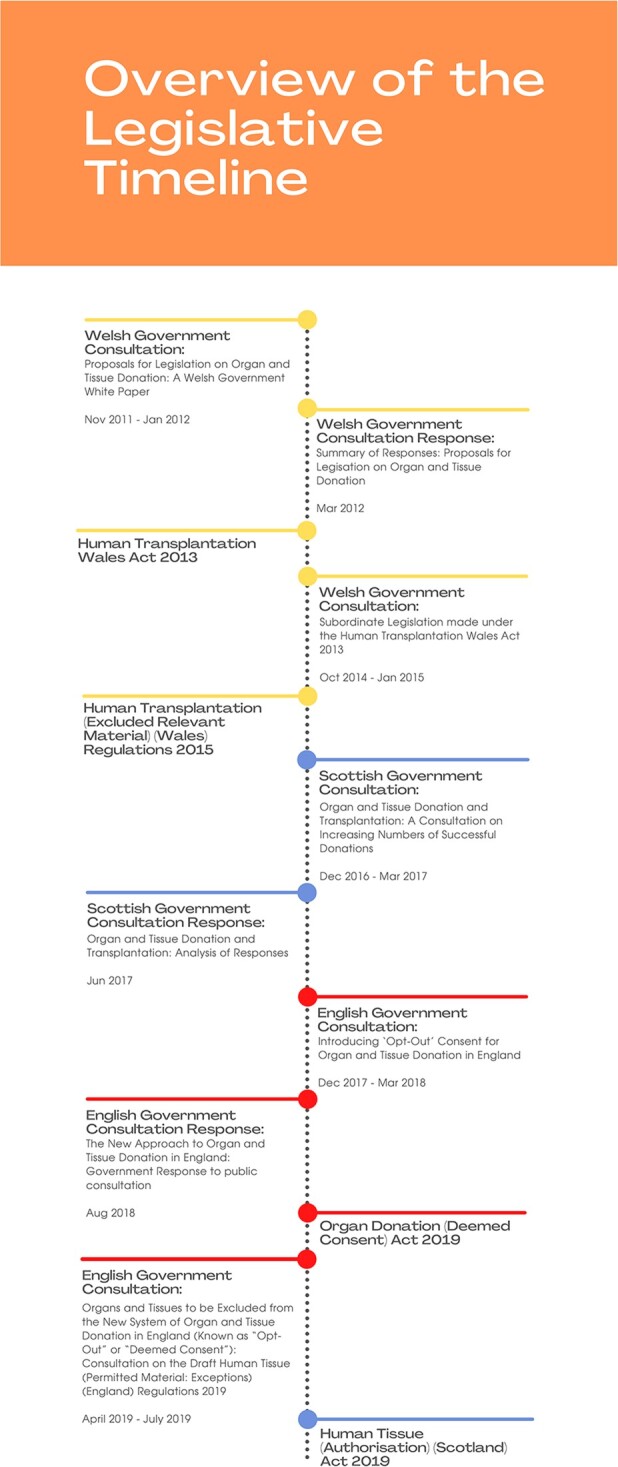

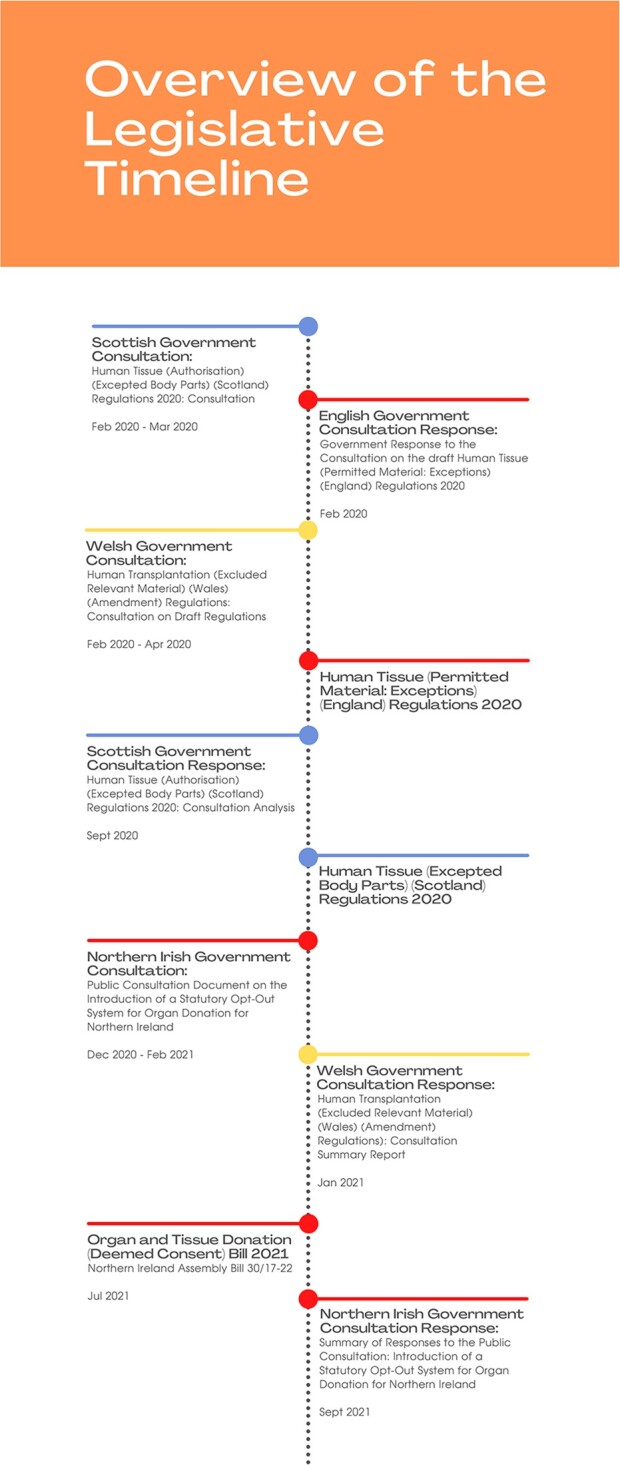
Policy timeline—the introduction of opt-out across the UK.

In all three countries, consultation exercises preceded both the creation of legislation governing deemed consent/authorisation,^100^ and the introduction of regulations specifying the excluded organs and tissues.^101^ Understanding how the approach of each country built on the experience of another not only serves to explain how and why England, Scotland, and Wales have arrived at particular sets of exclusions, but also illustrates the weaknesses inherent in the policy processes that led to their enactment. For a complete overview of the policy timeline see Fig. 1.

Perhaps unsurprisingly, given the emphasis placed by ministers separately in England, Scotland, and Wales on the importance of achieving legal consistency, regulations specifying organ and tissue exclusions bear close resemblance to one another, or are the same in each country.^102^ Excluded from the opt-out systems and listed in the three sets of regulations are what each health department has referred to as ‘novel or rare transplants’.^103^ That is, all organs and tissues other than those that are commonly transplanted such as hearts, lungs, livers, corneas, skin, and bone. Thus, in order for the donation of novel organs and tissues to proceed, *explicit* consent must be provided by either the potential donor, their nominated representative, or qualifying relatives.^104^ Though a few minor differences exist in the substance and form of each regulatory approach, in the main, the effect of the regulations is the same, meaning that parity and therefore operational clarity within the transplant infrastructure is achieved. For example, regarding substance, in England and Wales the mouth and nose are listed for exclusion individually,^105^ whereas in Scotland these tissues are encompassed within the definition of the face^106^—the whole or any part of which is excluded from opt-out.^107^ Furthermore, in 2020 the Welsh government held a second public consultation on organ and tissue exclusions in order to amend the Welsh Regulations and bring them in line with those in England.^108^ To this end, it proposed specifying as ‘excluded relevant material’ eight further sexual and reproductive organs and tissues^109^ and cells used in Advanced Therapy Medicinal Products (ATMPs)—proposals with which most respondents to the consultation agreed.^110^ While Scottish Regulations do not cover ATMPs, the same sexual and reproductive organs and tissues excluded in the English Regulations are listed as ‘excepted body parts’.^111^

The situation in Northern Ireland is somewhat different because an opt-in system is currently still in operation. However, in December 2020 the Department of Health launched a 10 week public consultation on the introduction of a statutory opt-out system. This included both general questions regarding the implementation of any opt-out policy in Northern Ireland, and broad questions related to the scope of the policy (for example, whether certain demographic groups and novel and rare types of donation should be exempt or excluded from the new legislation meaning explicit consent to donation would still be required).^112^ In September 2021, the Northern Irish Minister for Health announced that in the light of strong public, professional, and voluntary sector support for consultation proposals, the Department of Health would proceed with a draft bill—the Organ and Tissue Donation (Deemed Consent) Bill—which, having passed through all stages of the Northern Ireland Assembly, now awaits Royal Assent.^113^

While it appears that the Northern Irish opt-out system will only apply to common and routine transplants,^114^ the proposed form of the regulations to govern the new system differs substantially from regulations in the rest of the UK. Indeed, in its summary of responses to the public consultation, the Northern Irish Department of Health confirmed that ‘rather than prescribing lists of exempt organs, NI Regulations will explicitly state the organs to which deemed consent will apply'.^115^ This means that all organs and tissues falling outside of the regulations will not be covered by any new opt-out law and will continue to require explicit consent for donation to lawfully proceed. As opt-out legislation has not yet been enacted in Northern Ireland and the Northern Irish Government is yet to consult the public on the specific question of organ and tissue inclusions or exclusions, it is not clear what exactly that will include but it seems likely, for the reasons articulated below, that the scope of the opt-out system will be the same as in the other nations in the UK.

### B. Rationales Underpinning Organ and Tissue Exclusion Policies across the UK

As noted in Section III above, restrictions on the scope of new opt-out laws should, in the absence of reasons to the contrary, be driven by the same rationale(s) that underpinned the shift to opt-out in the first place. Across the UK, reform of organ donation and transplantation laws has been pursued with two key goals in mind: increasing the supply of organs and tissues available for transplantation and better reflecting PMOD preferences. In the English, Scottish, and Welsh policy documents proposing organ and tissue exclusions, and in the parliamentary debates on the passing of relevant supporting legislation, both of these goals are either explicitly or implicitly referenced as providing the underlying rationale for the proposed (now accepted) organ and tissue exclusions^116^ as discussed further below. However, as we discuss in Section V, whether the resulting regulations are best placed to achieve these goals is questionable.

#### 1. Increasing supply by maintaining trust and ensuring consistency

While it may be difficult to establish causative factors underpinning any increase in donation rates, it is clear from the policy statements highlighted in Section II(A) that this is seen as one of the main advantages of deemed consent organ donation systems.^117^ However, one matter that has the potential to adversely affect the achievement of this goal is the scope of deemed consent laws. Recognising this, politicians in England have expressed desires to avoid distress, and thus maintain public trust,^118^ by excluding novel and rare forms of transplantation from opt-out laws, given that the public may not expect these transplants to be included.^119^ This concern is centred on the assumption that including novel transplants within the opt-out system in England would be:

outside what the public would consider as common transplants … [and] not be consistent with the policy objective of changing the system in order to help those who are on a waiting list for a routine transplant.^120^

The concern here seems to be one articulated explicitly by Scottish policymakers—‘the unintended consequence of people deciding to opt out of donation due to a concern about donating a particular, rarely donated, body part’.^121^ To mitigate this risk, governments in England, Scotland, and Wales have, as discussed in Section IV(A), all restricted the organs and tissues in respect of which consent/authorisation may be deemed.

On the importance of maintaining public trust in systems of organ donation and transplantation, the Nuffield Council on Bioethics emphasises the central role that trust plays in creating and maintaining systems in which individuals are willing to donate and the potentially serious consequences for organ donation rates that medical mistrust may give rise to.^122^ Indeed, history (as discussed in Section III) has shown that public trust can easily be eroded. If this occurs, it can have potentially disastrous effects for donation rates, especially where public expectations and laws regulating organ donation are mismatched, and the law is considered to fail (deliberately or unintentionally) to respect the autonomous wishes of donors and/or donor families. By introducing blanket exclusions covering all organs and tissues which could be used in novel and rare transplants, governments thus seem to hope to reduce the likelihood of an Amiens-type scandal, resulting in significant loss of public trust and widespread registration of blanket refusals to donate, while still increasing the number of organs available for routine transplants.

Policymakers across the UK have also noted the potential for cross-border legal and/or policy differences in organ and tissue exclusions to complicate the donation and transplantation system, negatively affect the efficiency of current arrangements, and lead to confusion among both medical professionals and relatives of the deceased, resulting in a loss of public trust in organ donation.^123^ Across the UK, organ and tissue donation is coordinated by one central body—NHSBT.^124^ This is so notwithstanding differences in the consent provisions for organ donation between England, Scotland, Wales, and Northern Ireland. That is, once consent/authorisation has been either deemed (in England, Wales, and Scotland) or explicit consent has been given (in respect of all organs and tissues in Northern Ireland until opt-out law is enacted and in respect of all excluded organs and tissues in England, Scotland, and Wales), organs and tissues can be allocated to a patient in any part of the UK.^125^ Given this, all three public consultations on organ and tissue exclusions referred to the importance of achieving broad consistency^126^ or ‘parity’.^127^ Such a consistent approach enables healthcare professionals working across borders to come to a common understanding on the organs and tissues that constitute routine transplants (for which consent/authorisation may be deemed) which is said to be reassuring to patients and their families,^128^ and thereby ensures that the UK-wide system of organ donation and transplantation operates effectively.^129^

#### 2. Reflecting prospective donors post-mortem donation preferences

As explained in Section II(B), the second major policy rationale underpinning shifts to opt-out donation systems across the UK has been that of delivering closer alignment between organ retrieval practices and the preferences, wishes, and values of potential organ donors prior to their deaths. This has clearly influenced the approach taken to determining relevant organ and tissue exclusions across the UK, with governments in England, Scotland, and Wales each undertaking public consultation exercises focussed on proposed organ and tissue exclusions. In England, the consultation exercise sought views on whether respondents ‘agree[d] with the Government’s proposed list of excluded transplants’,^130^ and provided a tick box exercise listing proposed exclusions and asking ‘which … should be excluded from opt-out’.^131^ In Scotland, views were sought on whether there were ‘any parts of the body [in particular listed groups] that should not be listed’,^132^ or ‘if there is anything that is missing’.^133^ And in Wales, the second consultation on excepted body parts asked whether participants ‘agree[d] with the [Government’s] proposed new additions to the … regulations’.^134^ By consulting the public about which organs and tissues should continue to require express consent, policymakers clearly sought to ascertain individuals’ selective preferences in relation to all non-routine transplants with the aim of utilising responses to guide and shape the introduction of statutory organ and tissue exclusions.

Indeed, in response to the consultations, and to reflect comments received from the public, each health department either made further changes to the proposed regulations, for example, by adding additional organs and tissues to lists of exclusions^135^ and revising the definition of a particular structure contained in the regulations,^136^ or confirmed its intention to proceed with introducing the proposed regulations as a draft bill for approval where most respondents agreed with the draft proposals.^137^ Thus, the regulations approved in England, Scotland, and Wales constituted genuine attempts to reflect people’s PMOD preferences. How successful those were, however, is open to question, and we explore this further below.

## V. CRITICAL ASSESSMENT OF ORGAN AND TISSUE EXCLUSION LAW AND POLICY DOCUMENTS

In what follows, we advance several criticisms of the approaches taken in England, Scotland, and Wales to the formation and amendment of organ and tissue exclusion law and policy, and put forward recommendations for improvement. We focus on four issues. First, the design and framing of the consultations; in particular, the approach taken towards evidence, the timing and duration of the consultations, and the audiences targeted in the consultation exercises. Secondly, the information provided to the public during the consultation exercises regarding transplantation possibilities and purposes. Thirdly, we consider the inability to record selective donation preferences in relation to excluded organs and tissues on the ODR. Finally, we examine the proposed process and criteria by which organs and tissues excluded from presumed consent schemes may be amended.

### A. Evidence, Timing, and Target Audience

One significant flaw with the English, Scottish, and Welsh consultations on organ and tissue exclusions was that the initial proposed lists of exclusions were not evidence based. For example, no robust studies into donation preferences were cited (such as, the quantitative and qualitative evidence outlined in Section III(A)) as being used to inform the list of exclusions initially proposed by Wales, the model on which the approaches in England and Scotland were initially based. Given the paucity of responses to the first Welsh consultation about transplant exclusions, 17 responses were received,^138^ the current Welsh Regulations reflect what the Welsh Government *anticipated* beliefs about organ donation to be, without a reliable supporting evidence base.

Having said that, consultations themselves can be evidence-gathering tools of a sort and, in this context, they were used to inform and revise further amendments to draft sets of regulations after the consultation responses had been analysed. However, that the *initial* proposed lists of organ and tissue exclusions offered to the public were not evidence-based remains problematic—especially as those initial lists may, in turn, have biased the consultation responses (for example, due to status quo bias and framing effect).^139^ It would, therefore, have been better (assuming that the consultation process *was* a genuine attempt to gather evidence) for a single comprehensive list of all common and novel/rare organs and tissues for transplantation to have been presented. This would have allowed respondents to the consultations to select tissues for inclusion or exclusion themselves, and avoided the risk of biasing the consultation outcome towards the list presented.

With respect to the timing of the consultations on organ and tissue exclusions across the UK, those in Scotland and Wales were launched against the backdrop of the SARS-CoV-2 (2019-nCoV) coronavirus 2019 (COVID-19) pandemic. The Scottish consultation ran for six weeks from February to March 2020,^140^ and the Welsh consultation for nine weeks from February to April 2020.^141^ This is problematic for obvious reasons. At the time of these consultations, the threat posed by the virus was beginning to emerge. On 19 March 2020, the Coronavirus Bill setting out measures to respond to the COVID-19 outbreak, was introduced in the House of Commons and rapidly advanced through Parliament to receive Royal Assent on 25 March 2020.^142^ On 23 March 2020, the Prime Minister announced a series of UK-wide lockdown measures to try to contain the spread of the virus.^143^ Given the strong likelihood that most people were preoccupied by the evolving COVID situation, it is reasonable to assume that the majority of individuals were either less aware that the Scottish and Welsh governments were seeking views on proposed initial and additional transplant exclusions, or were less inclined to participate due to more immediate and pressing concerns. Neither government could have predicted the disruption to public and political life that has gone on to occur due to the pandemic. However, once the severity of the threat to public health was established and various measures to control the spread of the disease were introduced, decisions should have been made to either suspend or extend the duration of the consultations to give the greatest number of people the chance to engage with the relevant issues.

A further shortcoming of the Scottish and Welsh consultations was the decision to run them for only six and nine weeks respectively, contrary to the usual recommendation that consultations last for at least 12 weeks.^144^ These time limits ran counter to the stated aims of designing and implementing policy which respects the wishes of prospective organ donors and reduces the likelihood of opt-outs from those who object to the donation of rare or novel tissues.^145^ Compared to the 12-week English consultation which ran from 29 April to 22 July 2019,^146^ the shorter duration of the consultations in Scotland and Wales means that fewer people may have had the chance to consider the relevant issues and participate, and the quality of responses may have been reduced as a result.^147^

A final concern about the Scottish consultation is that, unlike its Welsh and English counterparts, it emphasised that views were ‘primarily sought from the clinical community who have experience of the deceased donation and transplantation pathway, and their representative organisations and bodies’.^148^ This was problematic for two reasons. First, deemed authorisation laws and accompanying regulations apply to all capacitous adults ordinarily resident in Scotland for a period of at least 12 months prior to their death.^149^ As such, *all* those affected by the new deemed authorisation policy should have been encouraged to take an active role in informing the development of the law by expressing their preferences in relation to proposed exclusions. Secondly, as noted above, the consultation was launched during the early stages of the pandemic when the clinical community, in particular, was under significant pressure. For example, on 17 March 2020, the NHS Chief Executive and the Chief Operating Officer jointly released a letter setting out important actions that every part of the NHS was asked to put in place, including redirecting staff and resources and building on actions such as freeing up the maximum possible inpatient and critical care capacity.^150^ Aiming the consultation at a limited and already overburdened target audience may mean that engagement was lower than it otherwise might have been.

Additionally, in 2016, the Scottish Government had included a question about what provisions should apply to the less common types of organs and tissues in its broader three month consultation targeted at the general public on moving to an opt-out system of organ donation.^151^ However, participants were only provided with two tick box options in response: either that deemed authorisation should apply to the more common organs and tissue, or that it should apply to *all* organs and tissue.^152^ As such, opportunities for the public and the clinical community to engage with this important policy issue were seriously compromised. The extent to which the Scottish regulations reliably reflect preferences is, therefore, uncertain.

### B. Information Provision

If regulations excluding certain organs and tissues from a policy of ‘deemed consent’ are to be based, in part, on evidence of the public’s beliefs, consultation participants should have been provided with up-to-date information about current transplant possibilities to make truly informed choices. However, no accompanying information about donating novel or contentious organs and tissues, or the purpose and clinical feasibility of their transplantation was provided in the English, Scottish, or Welsh consultations, alongside proposed lists of excluded material.^153^ In the English context but not in the Scottish or Welsh consultations, various examples of public confusion and misinformation regarding current transplantation practices with respect to excluded organs and tissues are evident in the Government response published by the DHSC.^154^ For instance, the response observes that ‘many [respondents] expressed concern about the potential transplantation of reproductive organs and tissues and asked for clarity on the situation with embryos and foetuses’.^155^ To clarify, the Government went on to explain that it was not (currently) technically possible to transplant an embryo and that any removal of an embryo would almost certainly destroy the embryo itself.^156^ Explaining why an embryo inside the body had been proposed as a specific exclusion, the Government response states that it was added ‘to put beyond doubt that together with other reproductive tissues and sexual organs, it will not be covered by deemed consent.’^157^ Further confusion also arose regarding the prospect that sperm and eggs may be transplanted with testicles and ovaries.^158^ Explaining the current regulatory regime, the Government confirmed that gametes fall under the remit of the Human Fertilisation and Embryology Act 1990 (as amended), and that if transplantation technology develops to make the transplant of donor ovarian and testicular tissue possible, appropriate mechanisms would need to be established to ensure appropriate regulation.^159^

As these examples demonstrate, members of the public are likely to lack specialist knowledge about transplantation and are thus unlikely to know which organs and tissues it is possible to transplant, the purpose of different transplants, and the frequency with which they occur. In the countries with opt-out donation systems in the UK, draft proposals for excluded transplants have now passed into law. However, in the future, should policymakers seek to propose additional transplants for exclusion (as seen in Wales),^160^ or propose amendments to existing regulations to remove an organ or tissue from the list so that consent may be deemed, they should also ensure that the public is provided with sufficient and accurate information.

### C. Recording Donation Preferences for Organs and Tissues Excluded from Opt-Out Systems

In the three constituent parts of the UK with systems of opt-out donation, various policy documents state that organs and tissues not covered by deemed consent or deemed authorisation (that is, novel and rare organs and tissues) may only be removed, stored, or used for transplantation with the explicit consent of the potential donor prior to their death, or their nominated representative, or family members after death.^161^ However, disappointingly, in all of these systems, individuals will not have an opportunity to express their donation preferences in relation to these excluded organs and tissues by recording such preferences on the NHS ODR. Although England, Scotland, and Wales have each implemented opt-out systems, the ODR remains an important source of evidence of donation preferences. The ODR website not only provides people with the opportunity to register a decision that they do not wish to donate after death, that is to opt out, but also continues to enable people to register a decision that they would like to donate some or all organs and tissues. As part of this process, individuals are provided with the opportunity to express their donation preferences in relation to the organs and tissues falling within the scope of opt-out schemes (such as heart, lungs, or kidneys) by selecting which of these they would like to donate.^162^

However, while consent to the donation of organs and tissues for novel and rare transplants must be explicitly provided, as consent was provided for all forms of donation in the previous opt-in systems, there is no opportunity for the *willing* donor of novel or rare tissues to record such a preference. The willing uterus or limb donor, therefore, cannot make their preferences known on the official donor register. Instead, where there is demand for such novel organs and tissues, consent will likely be sought from the deceased’s family members or nominated representative. In England, for example, the DHSC has confirmed that:

if you die in a hospital that runs a novel transplant programme, you are a suitable donor and there is someone on a waiting list for such a transplant, your family will be asked whether you expressed a decision to donate your organs, tissues and cells for novel transplants. Their consent will be sought to go ahead with a novel transplant, if this is a possibility.^163^

This entails asking questions to ascertain whether the proposed donor ‘expressed a decision to donate [their] organs, tissues, and cells for novel transplants’,^164^ and/or whether they ‘would have been unwilling for the excepted part to be removed for transplantation purposes’.^165^ But relying on information from families, and the failure to even attempt to record preferences regarding excluded organs and tissues, are problematic. It is well known, for example, that many prospective organ donors will not have discussed organ donation preferences with family members prior to death.^166^ Indeed, as proposed lists of exclusions from deemed consent systems may contain more than 30 specified organs, tissues, and cells,^167^ very few of those who have discussed their organ donation preferences with family members will have discussed the complex issues raised by those specific tissues and, more generally, by novel, rare, or experimental forms of transplantation. Furthermore, it is well recognised that family members are imperfect proxies, and may find it difficult to both ‘don the mental mantle’^168^ of their relatives and separate their own views and preferences regarding organ donation from those of the family member they represent.^169^

Given this, allowing donation preferences in respect of excluded organs and tissues to be recorded on the ODR prior to death would enable a more accurate account of those preferences and would better align with the underlying ethos of ‘appropriate consent’ in the Human Tissue Act 2004 and ‘express authorisation’ in the Human Tissue (Scotland) Act 2006. It would ensure that preferences regarding *all* organs and tissues were officially recognised and would better satisfy the preferences of those who, in signing the ODR, tick the newly included box stating that they do not want NHS staff to speak to their family regarding ‘how organ donation can go ahead in line with [their] faith or beliefs’.^170^ Note, however, that this does not mean that family members will not be consulted about the decision to donate their relative’s organs. In fact, in practice, as noted in the recent parliamentary debates on the passing of the English Regulations, ‘the involvement of the family in discussions about organ donation will remain absolutely a paramount consideration’.^171^ Any change to the ODR to encompass a more comprehensive range of organs and tissues (including routine *and* novel and rare transplants) is, of course, something that would need to be taken forward on a UK-wide basis to ensure parity, fairness, and the smooth operation of the donation system across borders. We thus suggest that NHSBT explores the possibility of recommending that people should be able to record their donation preferences on the ODR with respect to any organs and tissues whether within the scope of opt-out systems or not. As ODR records play an important role providing evidence of an individual’s PMOD wishes with respect to routine organs and tissues, so too would such records play this role regarding the donation and transplantation of novel organs and tissues. Indeed, as discussed previously, it may be harder for those in a qualifying relationship or nominated representatives to ascertain these preferences in the absence of such direct evidence.

### D. Revision of Current Exclusions from Opt-Out Systems

Finally, questions may be raised about whether the criteria set out for considering revisions to the lists of organs and tissues excluded from opt-out systems and the process for amending the regulations, align with the two principal goals that shifts to opt-out are designed to achieve. The position explicitly adopted in England, and mirrored in Scotland and Wales, is that:

if a novel transplant became standard practice and there was high demand for transplants of that organ or tissue, the Government would consider removing it from the list of organs and tissues excluded from opt-out.^172^

There are clearly practical reasons to reconsider exclusions once novel transplants become commonplace and/or demand for previously rare transplants can no longer be met through express consent (of either donors prior to their deaths or their family members or nominated representatives post-mortem). Decisions, however, to remove organs and tissues from lists of exclusions from opt-out systems, should, as at the time of an original decision to exclude an organ or tissue, be clearly set out in policy documents. They should also be based on empirical data and reasoned assumptions regarding the donation preferences of potential donors, as well as considerations relating to the maintenance of public trust in organ donation. This approach would provide for better alignment between policy and the informed preferences of the public, and would help to avoid causing unnecessary distress to the family of the deceased (and related public scandals and mass opt-outs) in the event that unexpected organs and tissues are retrieved absent explicit consent. This, however, has not been the case, and policy documents across the UK note only high demand and entry into standard practice as criteria for reconsideration of exclusions.

As to the process for amendments, changes to current exclusions, beyond updates to achieve parity between countries,^173^ are not anticipated in the near future in the UK;^174^ nevertheless, relevant revision processes have been mapped out. The most detailed of these has been provided by the DHSC and has three stages.^175^ First, the Government will consult with relevant public bodies including ‘NHSBT, NHS England, clinicians and any other relevant clinical stakeholders’.^176^ Draft regulations will then be laid before Parliament for debate and approval.^177^ Finally, if approval is granted, a Written Ministerial Statement explaining ‘why the change has been made and the impact it will have’, would be issued.^178^ A similar (though less explicit) amendment process has been proposed for Scotland,^179^ and such a process was recently undertaken in Wales when the public were consulted on proposed additions to the 2015 Regulations.^180^

These processes for amendments are to be tentatively welcomed as they signal the involvement of key stakeholders including organisations with expert knowledge, and governmental and parliamentary scrutiny of proposed legislative changes. However, while we suggest that the public should be viewed as an ‘other relevant clinical stakeholder’, a commitment to engaging with members of the public is not explicitly stated in any relevant policy documents. Given the goals of increasing the supply of organs available for transplantation and reflecting PMOD preferences, governments should explicitly commit to engaging the public, particularly in the event that proposals to remove excluded organs and tissues contained in current regulations are advanced.

## VI. CONCLUSION

The arguments for and against opt-out systems for PMOD have been discussed at length in the academic literature and beyond. Far less attention, however, has been given to the question of what the *scope* of opt-out systems should be. This article goes some way towards plugging that gap by asking how governments should decide which organs and tissues are within scope of opt-out organ donation systems (such that active consent is no longer needed) and which out of scope (meaning that active consent is still required—from the donor prior to death, or from a third party with legal authority).

We have used recent (and ongoing) shifts to opt-out organ donation systems in England, Scotland, Wales, and Northern Ireland (proposed), as lenses through which to examine this scope question. Our analysis reveals that, in common with many other jurisdictions outside the UK, two main policy goals underpinned shifts to opt-out systems: increasing the supply of organs and tissues for transplant, and more closely aligning transplantation outcomes after death with people’s preferences in life. Specifically, opt-out systems more readily enable organs to be taken from those who wanted to donate but did get around to joining to the ODR during their lifetimes.

We have argued that to make the overall regulatory framework as coherent and effective as possible, the considerations that generated the original move to an opt-out system should also determine the scope of that system. This means that we should be looking to maximise both the supply of organs and tissues available for transplantation and the extent to which PMOD outcomes are aligned with people’s preferences. To meet these objectives, regulations setting out the scope of opt-out schemes need to be based on the best available social science evidence regarding people’s PMOD preferences, and in particular, preferences surrounding the donation of different body parts. For example, that people are more positive about donating kidneys than faces or uteri would be highly relevant and could justify the latter not being within the scope of an opt-out system. While, on the face of it, leaving some organs and tissues outside the scope of such systems conflicts with the goal of increasing supply, we have argued that there are often indirect reasons why the opposite is the case. Foremost among these is that leaving the organs and tissues required for controversial, experimental, or rare transplants outside of the scope of opt-out donation systems (thus presuming dissent in the absence of appropriate consent to donate) may reduce negative effects on public sentiment and trust.

Having outlined an overall framework for how governments should approach scope questions, the article returns to its central case study. It critiques the policy formation processes in England, Scotland, Wales, and Northern Ireland, and makes recommendations for improvement, many of which are generalisable to other countries. We found that the evidence-gathering attempts on which policy formation was based in the UK often fell short, and that the public consultation exercises were similarly lacking in rigour, with many of the methods deployed liable to generate biased or otherwise unreliable information. In addition, across the UK, it seems that individuals will regrettably not have an opportunity to explicitly consent to donate, via the NHS ODR, the excluded organs and tissues contained in the three sets of regulations. They will be able to express and have recorded on the NHS ODR their views on donating those organs and tissues that are *within* the scope of the new opt-out policy (such as hearts and lungs), but will not have the same chance to do so for those *outside* its scope (such as hands or uteri). If alignment of transplantation outcomes with people’s preferences is genuinely one of the goals of the system, this should be rectified.

In summary, our key recommendations for policymakers designing opt-out systems are as follows. First, at a fundamental level, decisions about the scope of any such system (for example, to which organs and tissues does it apply?) should always be driven by the very same considerations that were taken to motivate and justify the original preference for an opt-out system. Secondly, these motivating considerations are highly likely to contain two core elements: a desire to improve the availability of transplant organs, and a desire to align PMOD outcomes more closely with people’s preferences while alive. Third, these two aims may sometimes *seem* at odds with one another because respecting individual preferences regarding PMOD can place constraints on the utilisation of organs. However, opt-out systems that are well aligned with the population’s views on organ donation will typically also be more efficient from an availability of organs point of view. This is because of the positive effect that such alignment has on public attitudes towards the organ donation system; or, to put it another way, the absence of alignment has potentially corrosive effects on public trust which could ultimately reduce the availability of organs by encouraging opting-out. Finally, the public’s views on organ donation cannot be just assumed. Consequently, those tasked with designing and implementing opt-out systems must seek out and act on the best available evidence about public attitudes and ensure that any consultation exercises are evidentially robust and unbiased.

## Funding

This research was supported by The Leverhulme Trust (grant no: ECF-2018-113) and the Wellcome Trust (grant no: 097897/Z/11/Z).

*Conflict of interest statement*. None declared. 

The authors would like to thank the two anonymous reviewers and an editor of this journal who provided helpful and constructive comments on earlier drafts of this article. We also express our gratitude to attendees of the *2021 Ethox seminar series*, the *2019 Ethics and Regulation of Reproductive Donation Conference*, the *2018 European Society of the Philosophy of Medicine and Healthcare Conference*, and the *2018 International Association of Bioethics Congress*, who provided excellent comments on parts of this article. This research was supported by The Leverhulme Trust (ECF-2018-113) and the Wellcome Trust (097897/Z/11/Z).

## Footnotes

1 See, eg: RH Thaler and CR Sunstein, *Nudge: Improving Decisions about Health, Wealth, and Happiness* (Penguin 2009) 184–92; Department of Health and Social Care (DHSC), ‘Consultation on Introducing “Opt-Out” Consent for Organ and Tissue Donation in England’ (2018) <https://www.gov.uk/government/consultations/introducing-opt-out-consent-for-organ-and-tissue-donation-in-england/consultation-on-introducing-opt-out-consent-for-organ-and-tissue-donation-in-england> accessed 28 November 2020; British Medical Association, ‘Parliamentary Brief: Organ Donation (Deemed Consent) Bill 2017-19’ (British Medical Association 1 February 2019) <https://www.bma.org.uk/media/1423/bma-briefing-on-organ-donation-deemed-consent-bill-hoc-in-england-feb-2019.pdf> accessed 16 November 2021.

2 For an exploration of the key differences between different ways of characterising the authorisation aspect of opt-out policies (eg as ‘presumed’ or ‘deemed’ consent), see B Saunders, ‘Opt-out Organ Donation Without Presumptions’ (2012) 38 J Med Ethics 69.

3 The term ‘donate’ is placed in scare quotes here in recognition of the fact that legitimate questions can be raised regarding whether organ retrieval from the deceased absent explicit consent can accurately be described as an act of donation. Donation, after all, is a form of gifting, and it is generally assumed that only items willingly given should be considered gifted. Thus, those who consider opt-out organ donation policies to constitute ‘presumed’ consent rather than ‘deemed’ or ‘tacit’ consent, may well object to the use of ‘consent’ here. For this audience, please feel free to replace ‘donation’ with ‘retrieval’ where appropriate.

4 EJ Johnson and D Goldstein, ‘Do Defaults Save Lives?’ (2003) 302 Science 1338, 1338; CR Sunstein, ‘Deciding by Default’ (2013) 162 U Pa L Rev 1, 23; CRM McKenzie, MJ Liersch and SR Finkelstein, ‘Recommendations Implicit in Policy Defaults’ (2006) 17 Psychol Sci 414.

5 TM Wilkinson, *Ethics and the Acquisition of Organs* (Clarendon Press 2011) 95; Thaler and Sunstein (n 1).

6 For examples, see: Thaler and Sunstein (n 1); V English, ‘Is Presumed Consent the Answer to Organ Shortages? Yes’ (2007) 334 BMJ 1088; A Caplan, ‘Organ Transplants: The Costs of Success’ (1983) 13 Hastings Cent Rep 23; MB Gill, ‘Presumed Consent, Autonomy, and Organ Donation’ (2004) 29 J Med Philos 37.

7 A Rithalia and others, ‘A Systematic Review of Presumed Consent Systems for Deceased Organ Donation’ (2009)13 Health Technol Assess 47; Human Transplantation (Wales) Act 2013 (HTWA 2013).

8 Organ Donation (Deemed Consent) Act 2019 (ODDCA 2019).

9 Human Tissue (Authorisation) (Scotland) Act 2019 (HTASA 2019).

10 The Organ Donation Act 2018 (Netherlands).

11 NIA Bill 30/17-22 Organ and Tissue Donation (Deemed Consent) Bill 2021.

12 Key examples of countries with ‘hard’ opt-out organ donation policies are Austria and Singapore: Federal law on the transplantation of human organs (OTPG) 2012 BGBI I Nr 108/2012, s 5(1) (Austria); Human Organ Transplant Act (Chapter 131A) 2012, s 5(2) (Singapore).

13 Key examples of opt-out countries where family members often play a significant role in authorising donation (as a result of legislative requirements and/or convention) are Belgium, Bulgaria, Croatia, Cyprus, Estonia, Finland, France, Greece, Hungary, Italy, Portugal, Slovak Republic, Slovenia, Spain, and Turkey. See J Costa-Font, C Rudisill and M Salcher-Konrad, ‘Relative Consent or Presumed Consent? Organ Donation Attitudes and Behaviour’ (2021) 22 Eur J Health Econ 5.

14 See, eg, H Carel, ‘The Problem of Organ Donation’ (2008) 42 Philos Mag 43; J De Wispelaere and L Stirton, ‘Advance Commitment: An Alternative Approach to the Family Veto Problem in Organ Procurement’ (2010) 36 J Med Ethics 180; TM Wilkinson, ‘Individual and Family Consent to Organ and Tissue Donation: Is the Current Position Coherent?’ (2005) 31 J Med Ethics 587.

15 See, eg, HTWA (n 9); ODDCA (n 8); HTASA (n 9); The Organ Donation Act 2018 (Netherlands).

16 England—Human Tissue (Permitted Material: Exceptions) (England) Regulations 2020, SI 2020/521 (HTPMEE Regulations 2020); Scotland—Human Tissue (Excepted Body Parts) (Scotland) Regulations 2020, SSI 2020/388 (HTEBPS Regulations 2020); Wales—Human Transplantation (Excluded Relevant Material) (Wales) Regulations 2015, SI 2015/1775 (W 247) (HTERMW Regulations 2015).

17 Thaler and Sunstein (n 1); Johnson and Goldstein (n 4) 1338; Sunstein (n 4) 23.

18 Thaler and Sunstein (n 1); McKenzie, Liersch and Finkelstein (n 4) 414–20; Sunstein (n 4) 23; D MacKay and A Robinson, ‘The Ethics of Organ Donor Registration Policies – Nudges and Respect for Autonomy’ (2016) 16 Am J Bioeth 3, 5.

19 The vast difference between organ donor registration rates in Austria (99%) and Germany (12%) is often cited as evidence of the power of the opt-out default in this context, given the nations’ shared history and cultural and linguistic similarities: JAC Everett and others, ‘Doing Good by Doing Nothing? The Role of Social Norms in Explaining Default Effects in Altruistic Contexts’ (2014) 45 Eur J Soc Psychol 230, 231.

20 A 2008 systematic literature review commissioned by the UK’s Organ Donation Taskforce, eg, found five studies (in three countries: Austria, Belgium, and Singapore) comparing donation rates pre and post transition to an opt-out system for organ donation, all of which showed (despite various shortcomings) increases in organ donation rates following the introduction of presumed consent legislation: Organ Donation Taskforce, ‘The Potential Impact of an Opt-Out System for Organ Donation in the UK—An Independent Report from the Organ Donation Taskforce’ (2008) <https://nhsbtdbe.blob.core.windows.net/umbraco-assets-corp/4250/thepotentialimpactofanoptoutsystemfororgandonationintheuk.pdf> accessed 8 October 2020, s 11.1; Rithalia and others (n 7) 20–24. Data available from Wales gathered between 2015 and 2017 also show a statistically significant increase in consent rates (from 45.8% to 61.0%) following the introduction of opt-out legislation: J Noyes and others, ‘Short Term Impact of Introducing a Soft Opt-Out Organ Donation System in Wales: Before and After Study’ (2019) 9 BMJ Open 1.

21 Organ Donation Taskforce (n 20) s 11.2.

22 ibid.

23 See, eg, P Michielsen, ‘Presumed Consent to Organ Donation: 10 Years’ Experience in Belgium’ (1996) 89 J R Soc Med 663, 663; LM Eleftheriou-Smith, ‘Organ Donation Set to Become Automatic for Dutch People Unless They Opt Out’ *The Independent* (London,14 September 2016) <https://www.independent.co.uk/news/world/europe/organ-donation-holland-automatic-dutch-unless-opt-out-death-a7307101.html> accessed 5 October 2021.

24 Welsh Government, ‘Consultation Document: Proposals for Legislation on Organ and Tissue Donation: A Welsh Government White Paper’ (WG13956) (2011) <http://www.wales.nhs.uk/sitesplus/documents/866/5.3b%20Organ%20and%20Tissue%20Donation%20-Consultation%20Document.pdf> accessed 5 October 2021, i.

25 V Young and others, ‘Welsh Government: Evaluation of The Human Transplantation (Wales) Act: Impact Evaluation Report’ (2017) (SRN:71/2017), p 1 <https://gov.wales/sites/default/files/statistics-and-research/2019-05/evaluation-human-transplantation-wales-act-impact-summary.pdf> accessed 8 October 2021.

26 Department of Health, ‘An Opt-Out System of Organ and Tissue Donation: Impact Assessment’ (2018), p 1 <https://assets.publishing.service.gov.uk/government/uploads/system/uploads/attachment_data/file/731915/Organ_donation_impact_assessment.pdf> accessed 8 October 2021.

27 Department of Health and Social Care (DHSC), ‘The New Approach to Organ and Tissue Donation in England – Government Response to Public Consultation’ (2018), 8 <https://assets.publishing.service.gov.uk/government/uploads/system/uploads/attachment_data/file/731913/govt-response-organ-donation-consent.pdf> accessed 8 October 2020.

28 Scottish Parliament, Human Tissue (Authorisation) (Scotland) Bill: Financial Memorandum (Scottish Parliament 2018), s 6 <https://archive2021.parliament.scot/S5_Bills/Human%20Tissue%20(Authorisation)%20(Scotland)%20Bill/SPBILL32FMS052018.pdf> accessed 5 October 2021.

29 SP Bill 32-PM Human Tissue (Authorisation) (Scotland) Bill [Policy Memorandum] Session 5 (2018), para 172.

30 Scottish Government, ‘Organ and Tissue Donation and Transplantation: A Consultation on Increasing Numbers of Successful Donations’ (2016) <https://consult.gov.scot/health-protection/organ-and-tissue-donation-and-transplantation/supporting_documents/00511160.pdf> accessed 5 October 2021, 3.

31 Human Tissue (Authorisation) (Scotland) Bill [Policy Memorandum] (n 29) para 12.

32 ibid.

33 ibid.

34 ibid para 171.

35 The stated ‘objective of the proposed change is to increase the current rate of consent for organ donation to proceed after a person’s death … increase the overall number of donors, and ultimately the number of lifesaving organs available for transplantation’: Department of Health (DH), Northern Irish Assembly (NIA), ‘Public Consultation Document on the Introduction of a Statutory Opt-Out System for Organ Donation for Northern Ireland’ (2020), p 6 <https://www.health-ni.gov.uk/sites/default/files/consultations/health/doh-organ-donation-consultation.pdf> accessed 11 January 2021.

36 DH, NIA, ibid 1.

37 ibid.

38 US Health Resources and Services Administration, ‘Organ Donation Statistics’ (2020) <https://www.organdonor.gov/statistics-stories/statistics.html> accessed 8 October 2020.

39 Transplant Australia, ‘The Facts’ (2020) <https://transplant.org.au/the-facts/> accessed 8 October 2020.

40 Australian Government, Organ and Tissue Authority, ‘2019 Australian Donation and Transplantation Activity Report’ (2020), 7 <https://donatelife.gov.au/about-us/strategy-and-performance/national-performance-data> accessed 8 October 2020.

41 DHSC (n 27) 8.

42 NHS Blood and Transplant (NHSBT|), ‘Organ Donation and Transplantation Activity Report 2019/20’ (2020), p 128 <https://nhsbtdbe.blob.core.windows.net/umbraco-assets-corp/19481/activity-report-2019-2020.pdf> accessed 8 October 2020.

43 It should be noted here that a methodological query can be raised regarding claims of a mismatch between public sentiment regarding organ donation and donor registration rates. For, should we read people’s preferences from what they say they prefer (in surveys tracking donation willingness), or from how they actually act (by registering or not registering to donate their organs and tissues)?

44 See, eg (n 4).

45 Welsh Government (n 24) 4.

46 DHSC, ‘The New Approach to Organ and Tissue Donation in England’ (n 27) 4. See also, Explanatory Notes to the ODDCA 2019, para 12.

47 DHSC (n 27) 7–8.

48 Human Tissue (Authorisation) (Scotland) Bill [Policy Memorandum] (n 29) para 6.

49 Scottish Government (n 30) 9.

50 DH, NIA (n 35) 9.

51 ibid 10.

52 For discussion, see, eg, Carel (n 14); De Wispelaere and Stirton (n 14); Wilkinson (n 14).

53 DHSC (n 1).

54 NHS Blood and Transplant (n 42) 132.

55 AL Caille-Brillet, R Zimmerling and HM Thais, ‘Bericht zur Repräsentativstudie 2018, Wissen, Einstellung und Verhalten der Allgemeinbevölkerung zur Organ- und Gewebespende’ BZgA-Forshungsbericht. Köln: Bundeszentrale für gusundheitliche Aufklärung (BZgA) 23, 32–33 (‘Report on the Representative Study 2018, Knowledge, Attitude and Behavior of the General Population towards Organ and Tissue Donation’, Cologne: Federal Centre for Health (BZgA)).

56 D Baughn, JR Rodrigue and DL Cornell, ‘Intention to Register as Organ Donors: A Survey of Adolescents’ (2006) 16.3 Prog Transplant 260, 264.

57 S Wöhlke, J Inthorn and S Schicktanz, ‘The Role of Body Concepts for Donation Willingness. Insights from a Survey with German Medical and Economics Students’ in RJ Jox, G Assadi and G Marckmann (eds), *Organ Transplantation in Times of Donor Shortage–**Challenges and Solutions* (Springer 2016) 27–50, 36; DB Sarwer and others, ‘Attitudes Toward Vascularised Composite Allotransplantation of the Hands and Face in an Urban Population’ (2014) 1 Vascular Compos Allotransplant 22, 26; US Department of Health and Human Services, ‘2019 National Survey of Organ Donation Attitudes and Practices: Report of Findings’ (2019), pp 21, 51 <https://www.organdonor.gov/sites/default/files/organ-donor/professional/grants-research/nsodap-organ-donation-survey-2019.pdf> accessed 5 October 2021.

58 S Wöhlke and others ibid.

59 DB Sarwer and others (n 57) 22–30, 26.

60 Health Resources and Services Administration, ‘2019 National Survey of Organ Donation Attitudes and Practices: Report of Findings’ (US Department of Health and Human Services, 2019). <https://www.organdonor.gov/sites/default/files/organ-donor/professional/grants-research/nsodap-organ-donation-survey-2019.pdf> accessed 5 October 2021, 21, 51.

61 NHS Blood and Transplant (n 42) Tables 4.4–4.6, 26–28.

62 A Newton (NHS Blood and Transplant Statistical Enquiries Service), Personal Communication, 26 November 2020.

63 L Excell and others (eds), ‘Australia and New Zealand Organ Donation Registry (ANZOD): Anzod Registry Report 2012’ (2012) <https://www.anzdata.org.au/report/anzod-annual-report-2012/> accessed 9 November 2020, 1–50.

64 MJ Dos Santos and others, ‘Trend Analysis of Organ and Tissue Donation for Transplantation’ (2020) 50 Transplant Proc 391.

65 LA Siminoff, RM Arnold and J Hewlett, ‘The Process of Organ Donation and Its Effect on Consent’ (2001) 15.1 Clin Transplant 39–47.

66 NHS Blood and Transplant Ocular Tissue Advisory Group, ‘Eye Procurement from Solid Organ Donors Activity Report 2016’ OTAG(16)32 (NHS Blood and Transplant 2016), pp 1, 5 <https://nhsbtdbe.blob.core.windows.net/umbraco-assets-corp/2790/eye_procurement_solid_organ_donors_june16.pdf> accessed 5 October 2021.

67 AL Caplan and others, ‘Moving the Womb’ (2007) 37 Hastings Cent Rep 18, 19; NJ Williams, ‘Should Deceased Donation be Morally Preferred in Uterine Transplantation Trials?’ (2016) 30 Bioethics 415; NJ Williams, ‘Deceased Donation in Uterus Transplantation Trials: Novelty, Consent, and Surrogate Decision Making’ (2018) 18 Am J Bioeth 18; M Freeman and PA Jaoudé, ‘Justifying Surgery’s Last Taboo: The Ethics of Face Transplants’ (2007) 33 J Med Ethics 76, 79.

68 Here, the concern expressed is not necessarily with the practice of organ retrieval from those who lack preferences, but with attempts to legitimise retrieval by reference to the mechanism of presumed/deemed consent. For, even though it is inappropriate to ‘deem’ or ‘presume’ consent in such circumstances, organ retrieval could, nevertheless, still prove justified on numerous other grounds.

69 For one of the earliest expressions of this concern, see RM Veatch, ‘Routine Enquiry About Organ Donation–An Alternative to Presumed Consent’ (1991) 325 N Engl J Med 1246, 1247–48.

70 J Miller, S Currie and RE O’Carroll, ‘If I Donate My Organs It’s a Gift, If You Take Them It’s Theft’ (2019) 19 BMC Public Health 1463, 1–15.

71 ibid.

72 ibid.

73 MacKay and Robinson (n 18) 3.

74 Ibid.

75 DHSC (n 27) 15.

76 C Csillag, ‘Brazil Abolishes “Presumed Consent” in Organ Donation’ (1998) 352 Lancet 1367.

77 T Patel, ‘France’s Troubled Transplant Trade: The Case of a Dead Teenager Whose Eyes Were Removed without His Parents' Consent Shocked the French Public. It Exposed a Transplant System Riddled with Legal and Ethical Failings’ *The New Scientist* (2 July 1993) <https://www.newscientist.com/article/mg13918802-700/> accessed 20 December 2021.

78 Organ Donation Taskforce (n 20) s 11.5.

79 P Tuppin and others, ‘National Census of Corneal Donations, Waiting Lists, and Transplantation in France’ (1997) 29 Transplant Proc 996, 996.

80 P Tuppin and others, ‘The Progress of Cornea Donation and Transplantation in France’ (1999) 18 Cornea 682, 682.

81 Thaler and Sunstein (n 1).

82 Human Tissue Act 2004 (HTA 2004) ss 2–3.

83 Human Tissue (Scotland) Act 2006 (HTSA 2006) ss 6–11.

84 HTA 2004, ss 1–3, 5–7; HTSA 2006, ss 6–10.

85 In England and Wales, individuals may nominate someone to make donation decisions on their behalf: HTA 2004, s 4.

86 HTWA (n 9) and ODDCA (n 8), which amend the HTA 2004.

87 HTASA (n 9), which amends the HTSA 2006.

88 Deemed consent legislation in England, Wales, and Scotland does not apply to minors or excepted adults; that is, those not ordinarily resident in England, Wales, or Scotland for a period of at least 12 months immediately before dying, or an adult who has died and who, for a significant period before dying, lacked capacity to understand deemed consent/authorisation provisions: HTWA 2013, ss 4–9; ODDCA (n 8), ss 1–2; HTASA (n 9), ss 5–9.

89 HTERMW Regulations 2015 (n 16); HTPMEE Regulations 2020 (n 16); HTEBPS Regulations 2020 (n 16).

90 S 16 amends the HTA 2004 to give effect to the new opt-out regime.

91 SI 2015/1775 (W 247).

92 HTWA 2013, s 4(6).

93 ODDCA (n 8) ss 1(4)–(5).

94 SI 2020/521.

95 ibid reg 2.

96 HTASA (n 9) s 7(2).

97 SSI 2020/388.

98 ODDCA (n 8), s 1(4).

99 HTA, ‘Code of Practice F: Donation of Solid Organs and Tissue for Transplantation – Part Two: Deceased Organ and Tissue Donation’ (2020) para 91 <https://content.hta.gov.uk/sites/default/files/2020-11/Code%20F%20part%202.pdf> accessed 22 December 2021.

100 DHSC (n 1); DHSC (n 27); Scottish Government (n 30); Welsh Government (n 24).

101 DHSC, ‘Organs and Tissues To Be Excluded from the New System of Organ and Tissue Donation in England (Known as “Opt-Out” or “Deemed Consent”): Consultation on the Draft Human Tissue (Permitted Material: Exceptions) (England) Regulations 2019’ (2019) <https://assets.publishing.service.gov.uk/government/uploads/system/uploads/attachment_data/file/798467/Organs_and_tissues_to_be_excluded_from_the_new_system_of_organ_and_tissue_donation_in_England_-_consultation_document.pdf> accessed 10 October 2021; Scottish Government, ‘Human Tissue (Authorisation) (Excepted Body Parts) (Scotland) Regulations 2020 Consultation’ (2020) <https://www.gov.scot/publications/consultation-human-tissue-authorisation-excepted-body-parts-scotland-regulations-2020/pages/2/> accessed 20 October 2021; Welsh Government, ‘Human Transplantation (Excluded Relevant Material) (Wales) (Amendment) Regulations: Consultation on Draft Regulations’ (2020) <https://gov.wales/sites/default/files/consultations/2020-02/consultation-document_0.pdf> accessed 1 October 2021.

102 HTERMW Regulations 2015, reg 2 (2)–(4); HTPMEE Regulations 2020, reg 2 (2)–(5), HTEBPS Regulations 2020, reg 2 (2)–(5).

103 DHSC (n 101); Scottish Government (n 101); Welsh Government (n 101).

104 HTA (n 99) paras 199–202.

105 HTPMEE Regulations 2020, reg 2 (2)(j) and (k); HTERMW Regulations 2015, reg 2 (2)(d) and (e).

106 HTEBPS Regulations 2020, reg 1 (2).

107 ibid reg 2 (2)(d).

108 Welsh Government (n 101).

109 These include the following: cervix, clitoris, fallopian tube, labia, vagina, vulva, prostate, and the perineum.

110 Welsh Government (n 101), 9–10.

111 HTEBPS Regulations 2020, reg 2 (2).

112 DH, NIA (n 35).

113 Northern Ireland Assembly, ‘Organ and Tissue Donation (Deemed Consent) Bill’ (2022) <http://www.niassembly.gov.uk/assembly-business/legislation/2017-2022-mandate/primary-legislation---bills-2017---2022-mandate/organ-and-tissue-donation-deemed-consent-bill/> accessed 9 February 2022; DH, NIA, ‘Summary of Responses to the Public Consultation: Introduction of a Statutory Opt-Out System for Organ Donation for Northern Ireland’ (2021) <https://www.health-ni.gov.uk/sites/default/files/publications/health/doh-response-and-analysis-to-the-organ-donation-soft-opt-out-consultation.pdf> accessed 28 October 2021; NIA Bill (n 11).

114 NIA Bill (n 11); ibid 17–18, 28–29.

115 ibid 25.

116 DHSC (n 1), DHSC (n 101); Scottish Government (n 30) Scottish Government (n 101); Welsh Government (n 24), Welsh Government (n 101).

117 ibid.

118 Organ Donation (Deemed Consent) Bill Deb 12 September 2018, col 4.

119 DHSC (n 101) 6.

120 ibid 10.

121 Scottish Government (n 101) 3.

122 Nuffield Council on Bioethics, ‘Response to the Government’s Consultation on Introducing “Opt-Out” Consent for Organ and Tissue Donation in England’ (2018) pp 1, 4 <https://www.nuffieldbioethics.org/wp-content/uploads/NCOB-response-to-consultation-on-introducing-‘opt-out’-consent.pdf> accessed 1 September 2021.

123 DHSC, ‘Government Response to the Consultation on the Draft Human Tissue (Permitted Material: Exceptions) (England) Regulations 2020’ (2020) <https://assets.publishing.service.gov.uk/government/uploads/system/uploads/attachment_data/file/867918/Government_response_to_opt-out_organ_donation_-_organs_and_tissues_excluded_from_the_new_system.pdf> accessed 28 October 2021, 14–15; Scottish Government (n 101) 5–6; Welsh Government (n 101) 6.

124 DHSC, ibid 14.

125 NHS Blood and Transplant (England) Directions 2005 (as amended) and NHS Blood and Transplant (Wales) Directions 2005 (as amended).

126 ibid 14–15; Scottish Government (n 101) 5.

127 Welsh Government (n 101) 6, 7, 9.

128 DHSC (n 123) 15.

129 ibid 14–15; Welsh Government (n 101) 6–7, 9; Scottish Government (n 101) 5–6.

130 DHSC (n 101) 13–16.

131 ibid.

132 Scottish Government (n 101) 10.

133 ibid.

134 Welsh Government (n 101) 11.

135 Explanatory Memorandum to the HTERMW Regulations 2015 <https://business.senedd.wales/documents/s500002735/CLA578%20-%20EM%20The%20Human%20Transplantation%20Excluded%20Relevant%20Material%20Wales%20Regulations%202015.pdf> accessed 9 October 2021, 4; DHSC (n 123) 16.

136 See the definition of the face contained in the proposed regulations in Scottish Government (n 101) compared with the HTEBPS Regulations 2020, reg 1(2).

137 Welsh Government, ‘Human Transplantation (Excluded Relevant Materials) (Wales) (Amendment) Regulations: Consultation Summary Report’ (2021) <https://gov.wales/sites/default/files/pdf-versions/2021/1/3/1610565538/human-transplantation-excluded-relevant-material-wales-amendment-regulations-consultation-summary.pdf> accessed 28 October 2021.

138 Explanatory Memorandum (n 135) 5.

139 A Tversky and D Kahneman, ‘The Framing of Decisions and the Psychology of Choice’ (1981) 211 Science 453.

140 Scottish Government (n 101) 11.

141 Welsh Government (n 101) 1.

142 UK Parliament, ‘Parliamentary Bills: Coronavirus Act 2020’ (2020) <https://bills.parliament.uk/bills/2731/stages> accessed 28 October 2021.

143 Gov.uk, ‘Prime Minister’s Statement on Coronavirus (COVID-19): 23 March 2020 (2020)’ <https://www.gov.uk/government/speeches/pm-address-to-the-nation-on-coronavirus-23-march-2020> accessed 28 October 2021.

144 Scottish Government, ‘Scottish Government Consultation Good Practice Guidance: Freedom of Information Release’ (2019) <https://www.gov.scot/binaries/content/documents/govscot/publications/foi-eir-release/2019/12/foi-201900009119/documents/foi-201900009119–-information-released/foi-201900009119–-information-released/govscot%3Adocument/FOI-201900009119%2B-%2BInformation%2BReleased.pdf> accessed 20 October 2021; Welsh Government, ‘Consultation Guidance for Staff, WG 10613’ (2014) <https://senedd.wales/deposited papers/dp-1431-11-16 welsh government staff consultation guidance to llyr gruffydd am from carwyn jones am in response-21052014-256427/dp-1431-11-16-cymraeg.pdf> accessed 2 July 2021, Principle E.

145 DHSC (n 101); Scottish Government (n 101); Welsh Government (n 101).

146 DHSC (n 101) 7.

147 Cabinet Office, ‘Consultation Principles 2018’ (2018) <https://www.gov.uk/government/publications/consultation-principles-guidance> accessed 27 October 2021.

148 Scottish Government (n 101) 1.

149 HTASA (n 9), s 7.

150 NHS England, ‘Important and Urgent–Next Steps on NHS Response to COVID-19’ (2020), pp 1–5 <https://www.england.nhs.uk/coronavirus/wp-content/uploads/sites/52/2020/03/urgent-next-steps-on-nhs-response-to-covid-19-letter-simon-stevens.pdf> accessed 28 October 2021.

151 Scottish Government (n 30) 18, 35.

152 ibid 35.

153 See DHSC (n 101); Scottish Government (n 101); Welsh Government (n 101).

154 DHSC (n 123) 10–21.

155 ibid 14.

156 ibid 15–16.

157 ibid 16.

158 ibid 14.

159 ibid 15.

160 Welsh Government (n 101).

161 Explanatory Memorandum to the HTPMEE Regulations 2020, para 2.2; Policy Note to the HTEBPS Regulations 2020, paras 8–9; Welsh Government (n 101) 7.

162 NHSBT, ‘Register Your Details–Yes I Donate’ (NHS Blood and Transplant, n.d.) <https://www.organdonation.nhs.uk/register-your-decision/register-your-details/?> accessed 20 October 2021.

163 DHSC (n 123) 29.

164 ibid.

165 HTASA (n 9), s 10.

166 NHSBT, ‘Families Need to Talk about Organ Donation, to Give Thousands Waiting the Chance of a “New Beginning”’ (2016) <https://www.organdonation.nhs.uk/get-involved/news/families-need-to-talk-about-organ-donation/> accessed 22 October 2021.

167 A total of 37 relevant materials are identified in the HTPMEE Regulations 2020.

168 TL Beauchamp and JF Childress, *Principles of Biomedical Ethics* (4th edn, Clarendon Press 1994) 171.

169 ibid; J de Groot and others, ‘Decision Making on Organ Donation: The Dilemmas of Relatives of Potential Brain Dead Donors’ (2015) 16 BMC Med Ethics 1; D Shaw and others, ‘Family Over Rules? An Ethical Analysis of Allowing Families to Overrule Donation Intentions’ (2017) 101 Transplantation 482.

170 NHSBT, ‘Register Your Details’ (n 162).

171 HC Deb 19 May 2020, vol 676, col 525.

172 DHSC (n 101) 10. Note that in Scotland, ‘high demand’ is expressed as ‘clinical demand’: Scottish Government (n 101) 4.

173 For example, as discussed previously, the Welsh Government recently consulted the public on updates to the Welsh regulations to avoid oversight and inconsistencies that became apparent in the light of the English regulations—see Welsh Government (n 101) 6.

174 DHSC (n 101) 10. See also DHSC (n 123) 10; Scottish Government (n 101) 4.

175 DHSC (n 101) 10.

176 ibid. Here we suggest that the wider public should be viewed as a relevant clinical stakeholder.

177 ibid.

178 ibid.

179 Scottish Government (n 101) 4–5.

180 See Section IV.

